# Probiotic Yeast Inhibits VEGFR Signaling and Angiogenesis in Intestinal Inflammation

**DOI:** 10.1371/journal.pone.0064227

**Published:** 2013-05-13

**Authors:** Xinhua Chen, Guoxun Yang, Joo-Hye Song, Hua Xu, Dan Li, Jeffrey Goldsmith, Huiyan Zeng, Patricia A. Parsons-Wingerter, Hans-Christian Reinecker, Ciaran P. Kelly

**Affiliations:** 1 Division of Gastroenterology, Beth Israel Deaconess Medical Center, Harvard Medical School, Boston, Massachusetts, United States of America; 2 Gastrointestinal Unit, Massachusetts General Hospital, Harvard Medical School, Boston, Massachusetts, United States of America; 3 Department of Pathology and Laboratory Medicine, Beth Israel Deaconess Medical Center, Harvard Medical School, Boston, Massachusetts, United States of America; 4 Department of Medicine, Division of Molecular and Vascular Medicine, Beth Israel Deaconess Medical Center, Harvard Medical School, Boston, Massachusetts, United States of America; 5 John Glenn NASA Research Center, Cleveland, Ohio, United States of America; Institute for Virus Research, Laboratory of Infection and Prevention, Japan

## Abstract

**Background and Aims:**

*Saccharomyces boulardii* (Sb) can protect against intestinal injury and tumor formation, but how this probiotic yeast controls protective mucosal host responses is unclear. Angiogenesis is an integral process of inflammatory responses in inflammatory bowel diseases (IBD) and required for mucosal remodeling during restitution. The aim of this study was to determine whether Sb alters VEGFR (vascular endothelial growth factor receptor) signaling, a central regulator of angiogenesis.

**Methods:**

HUVEC were used to examine the effects of Sb on signaling and on capillary tube formation (using the ECMatrix™ system). The effects of Sb on VEGF-mediated angiogenesis were examined *in vivo* using an adenovirus expressing VEGF-A(164) in the ears of adult nude mice (NuNu). The effects of Sb on blood vessel volume branching and density in DSS-induced colitis was quantified using VESsel GENeration (VESGEN) software.

**Results:**

1) Sb treatment attenuated weight-loss (p<0.01) and histological damage (p<0.01) in DSS colitis. VESGEN analysis of angiogenesis showed significantly increased blood vessel density and volume in DSS-treated mice compared to control. Sb treatment significantly reduced the neo-vascularization associated with acute DSS colitis and accelerated mucosal recovery restoration of the lamina propria capillary network to a normal morphology. 2) Sb inhibited VEGF-induced angiogenesis *in vivo* in the mouse ear model. 3) Sb also significantly inhibited angiogenesis *in vitro* in the capillary tube assay in a dose-dependent manner (p<0.01). 4) In HUVEC, Sb reduced basal VEGFR-2 phosphorylation, VEGFR-2 phosphorylation in response to VEGF as well as activation of the downstream kinases PLCγ and Erk1/2.

**Conclusions:**

Our findings indicate that the probiotic yeast *S boulardii* can modulate angiogenesis to limit intestinal inflammation and promote mucosal tissue repair by regulating VEGFR signaling.

## Introduction

*Saccharomyces boulardii* (*Sb*), a nonpathogenic probiotic yeast, has been used for decades to protect against intestinal injury and inflammation [1]. Animal and *in vitro* studies indicate that *S. boulardii* can protect against severe diarrhea and enterocolitis induced by a range of bacterial enteric pathogens including *Clostridium difficile*, *Vibrio cholerae, Salmonella typhimurium, Shigella flexneri*, enterohemorrhagic *E. coli* and enteropathogenic *E. coli* [1]–[7]. In human studies *S. boulardii* treatment significantly reduced the incidence of simple antibiotic-associated diarrhea, recurrent *C. difficile* diarrhea, and traveler’s diarrhea [8]–[13]. More recent clinical studies indicate that it may also be effective in inflammatory bowel disease (IBD) [14]–[17]. However, the mechanisms underlying the protective actions of Sb are not well understood.

Angiogenesis, the formation of new vasculature from an existing vascular network, is now recognized to play a critical role in various human disease processes, including carcinogenesis, tumor growth, and both acute and chronic inflammation [18]–[20]. There is considerable evidence *in vivo*, including clinical observations in humans, that abnormal angiogenesis is involved in many disease conditions including rheumatoid arthritis and other inflammatory disorders, carcinogenesis, psoriasisand ocular degenerative disorders including those seen in diabetes mellitus [18], [19], [21]. Recently, increasing clinical and experimental evidence indicates that angiogenesis also plays a crucial role in IBD [22]–[28]. Chronically inflamed intestinal tissues in IBD display significant alterations in microvascular physiology and function compared with healthy intestine and with uninvolved IBD intestine [27], [29].

Vascular Endothelial Growth Factor (VEGF) and the associated family of tyrosine kinase VEGF receptors (VEGFRs) are key proteins modulating angiogenesis [20], [30]. VEGFR types 1 and 2 (VEGFR-1 or Flt-1 and VEGFR-2 or KDR/Flk-1) are high-affinity transmembrane endothelial cell receptors for VEGF [30]. Of the primary receptors, VEGFR-2 is thought to mediate the majority of tumor angiogenic effects [19], [30]. Binding of VEGF to these receptors leads to intracellular receptor phosphorylation which activates various intracellular downstream signaling pathways leading to endothelial cell proliferation and blood vessel formation [18], [19], [31], [32].

We here demonstrate that Sb can regulate angiogenesis both *in vitro* and *in vivo*, by regulating the VEGFR activation and modulating capillary vessel formation during inflammatory responses thus promoting intestinal recovery from colitis.

## Materials and Methods

### Cells and Reagents

Human unbilical vein endothelial cells (HUVEC) were obtained from Clonetics. Cells were grown on plates coated with 30 µg/ml vitrogen in EGM-MV BulletKit (5% fetal bovine serum in endothelial basic medium (EBM) with 12 µg/ml bovine brain extract, 1 µg/ml hydrocortisone, 1 µl/ml GA-1000, and hEGF). HUVEC (passages 3 or 4) that are ∼80% confluent were used for most experiments. Cells were serum-starved in 0.1% fetal bovine serum in EBM for 24 h prior to testing. Antibodies against Erk, PLCγ, VEGFR2 phosphorylated and/or non-phosphorylated forms, were purchased from Cell Signaling Technology (Beverly, MA). Preparation of *Saccharomyces boulardii* culture supernatant (SbS) was performed as previously described [33], [34]. Briefly, lyophilized Sb (Biocodex Laboratories, France) was cultured in RPMI 1640 cell culture medium (100 mg/ml) for 24 hours in 37°C. The suspension was then centrifuged at 9000 g for 15 minutes and the supernatant collected. The supernatant was then passed through a 0.22 μm filter (Fisher Scientific) and then a 10 kDa cutoff filter (Millipore, MA).

### Western Blot Analysis

HUVEC were treated with VEGF (R&D Systems) with and without SbS at different time points. Treated cells were then lysed in a lysis buffer (62.5 mM Tris-HCl, 10% glycerol, 2% SDS, 0.01% bromphenol blue, and 1% 2-mercaptoethanol). Equal amounts of cell extract were fractionated by 4% to 20% gradient SDS-PAGE, and proteins were transferred onto nitrocellulose membranes (Bio-Rad) at 300 mA for 3 h. Membranes were blocked in 5% nonfat dried milk in TBST (50 mM Tris, pH 7.5, 0.15 M NaCl, 0.05% Tween 20) and then incubated with antibodies directed against phosphorylated and non-phosphorylated forms of VEGFR2, phopso-Erk1/2 and PLCγ. Membranes were washed with TBST and incubated with horseradish peroxidase-labeled secondary antibodies for 1 h. The peroxidase signal was detected by Supersignal chemiluminescent substrate (Pierce), and the image of the signal was recorded by exposure to x-ray film (Fujifilm, Tokyo, Japan).

### *In Vitro* Tube Formation Assay

ECMatrix™ assay kit (Millipore, Inc.) was used to study the effects of SbS on HUVEC capillary tube formation in accordance with the manufacturer’s instructions. HUVEC (∼1×10^4^ cells) were plated in 96-well plates previously coated with Matrigel and incubated in triplicates for 16 hours at 37°C in the absence or presence of SbS at different dilutions. Representative photomicrographs of tubule formation from 10 random fields from each group were captured. Tubular structures were then counted and expressed as the mean number of tubules expressed as a percentage of that counted in the control group.

### Mouse Ear Vasculature Assay

All animal protocols were approved by the BIDMC IACUC. Six-week-old, female, athymic, Nu/Nu mice (NCI, Bethesda, MD) were used in the mouse ear vasculature model as previously described.[35] A non-replicating adenoviral vector (Ad-VEGF-A164) engineered to express the predominant (164 aa) murine isoform of VEGF-A was a generous gift from Dr. Harold Dvorak. 5×10^6^ pfu of Ad-VEGF-A164 (in 10 µL) were injected into the dorsal skin of both ears using a 30-gauge needle. The first SbS (or vehicle) injection was administered s.c. locally (to the ear) 1 hour after the adenovirus injection. A second SbS (or vehicle) injection was administered 24 hours later. Ears were photographed on day 16.

### Angiogenesis Analysis in DSS-Colitis Model

DSS (4% for 5 days) was administered to 8-week-old female C57BL6 mice (Jackson Laboratory). Sb was given to mice daily by oral gavage at a dose of 6×10^8^ CFU. At Day 5, mice blood vessels were stained with 50 μg Alexa Fluor® 647 WGA (Molecular Probes) injected retro-orbitally. Seven minutes later, mice were sacrificed by CO_2_ asphyxiation. Colon tissues were immediately removed and rinsed with PBS. Tissue specimens were imaged with a Bio-Rad Radiance 2000 confocal microscope (Bio-Rad, Hercules, CA). Image acquisitions were carried out with Laser Sharp Scanning Software (Bio-Rad). Three dimensional analyses were used to assess the blood vessel volume and density in the mice colon. Three dimensional image reconstructions were translated into two dimensional grayscale images and binarized to black-and-white vascular patterns as described previously [36], [37]. The image of a binary vascular pattern was input into VESsel GENeration (VESGEN) software and upon selection of the Vascular Network analysis option. VESGEN automatically mapped and quantified the vascular pattern. The mapping of networks by VESGEN excludes any avascular spaces (AVS) located at the edge of the image because representation of the AVS is incomplete in those regions and therefore the vascular density is unknown.

### Histological Score Assessment of Colitis

H&E-stained colonic sections were coded for blind microscopic assessment of inflammation for DSS-induced colitis. Histological scoring includes parameters of colonic inflammation (ulceration, congestion, edema, and neutrophil infiltration) as previously reported [38].

### Statistical Analyses

Results were expressed as mean ± SE or mean ± STDEV. Data were analyzed using the SIGMA-STAT™ professional statistics software program (Jandel Scientific Software, San Rafael, CA). Analyses of variance with protected t test were used for intergroup comparison.

## Results

### Oral Intake of Sb Inhibits Angiogenesis during DSS Induced Colitis

**Figure 1:**
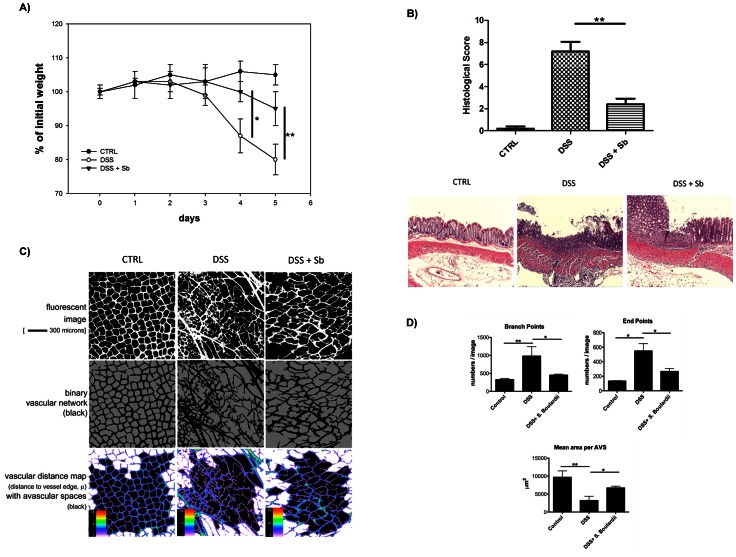
Oral administration of Sb reduced DSS-colitis induced weight loss, histological damage and neo-vascularization in mouse colon. We used the murine DSS-colitis model to assess the effect of Sb on neo-vascularization in acute colitis. We administered DSS (4% for 5 days) to mice to induce colitis. **1A)** Daily administration of Sb by gavage significantly attenuated weight-loss (N = 5, *p<0.05, **p<0.01). Data points represent mean relative weight ± standard error. **1B)** Histological scores for several parameters of colonic inflammation on Day 5 were evaluated [control (N = 5), DSS alone (N = 5), DSS plus Sb (N = 5)]. Data points represent mean score ± standard error, ^**^, p<0.01. Representative H/E stained colonic images are listed. **1C)** Confocal fluorescence images of the colonic microvasculature were taken 10 minutes after i.v injection of Alexa 647 WGA. Three dimensional image reconstructions were translated into two dimensional grayscale images and binarized to black-and-white vascular patterns for analysis using VESGEN software. Representative images are shown. **1D)** Quantitative data are presented in Tables 1 and 2. Comparisons of vascular network and avascular space indices among control, DSS and DSS+ Sb groups. Compared to control mice, the DSS group had significantly increased numbers of branch points and density of vessel endpoints (p = 0.004, p = 0.02 respectively). Sb treatment significantly reduced these DSS effects (p = 0.045, p = 0.02 respectively). The mean area per AVS (µm^2^) in the lamina propria was reduced in the DSS group compared to the control group (p = 0.01). Sb treatment normalized this effect (p = 0.02).

Dextran sulfate sodium (DSS) leads to reversible colitis with pathological angiogenesis in mice [22], [23], [28]. We therefore used this colitis mouse model to assess the effect of Sb on the remodeling of the colonic capillary vasculature during the induction of colitis. The luminal microvasculature of the normal intestine is organized as a highly regular ‘honeycomb’ lattice or network comprised of small diameter, capillary-like vessels (Figure 1C) surrounding the colon crypts. Three fundamental characteristics of such vascular networks are: (1) the vascular fractional area (or volume), (2) regularity of the geometry of the AVS as defined by network morphology or architecture and (3) a related characteristic, connectedness of the vascular network. The vascular fractional area is determined by vessel length and diameter in comparison to the fractional area of the avascular spaces (AVS’s). The relationship between the vascular and avascular fractional areas is inverse and the two fractional areas must sum to 1.0.

We administered DSS (4% for 5 days) to mice to induce colitis. As shown in Figure 1A, daily administration of Sb significantly attenuated weight-loss (N = 5, p<0.01) and reduced histological scores of inflammation (N = 5, p<0.01) caused by DSS administration (Figure 1B). Blood vessels were stained with Alexa Fluor® 647 WGA. Colonic tissue specimens were imaged and 3D reconstructions were completed.

The VESGEN network option analyzed the entire vascular pattern for vessel parameters (Figure 1C and Table 1), and the region containing complete AVS for network parameters (Figure 1C and Table 2). As shown in Figure 1C, the normal intestinal capillary network displays a highly regular lattice structure (left column) that was disrupted during severe inflammation caused by DSS and replaced by enhanced angiogenesis resulting in a vascular network characterized by increased numbers of branch and end points (right column and Tables 1 and 2). Sb treatment significantly reduced the number of capillary branch and endpoints indicating a reduced angiogenesis. Sb treatment also normalized vessel diameter and density in the colon lamina propria (middle column and Tables 1 and 2). Furthermore, the mean area per AVS (µm^2^) in the lamina propria was significantly reduced in the DSS group compared to control group (p = 0.01), whereas Sb again normalized the DSS effect (p = 0.01) (Figure 1D).

**Table 1:** Vascular network results (mean ± std dev).

Groups	Control	DSS	DSS+Sb
vessel diameter (μ)	16.4±0.5	17.6±0.7	17.4±1.4
fractional vasculararea	0.272±0.02	0.409±0.07	0.293±0.04
branch pointdensity (μ^−1^)	326±32	981±259	452±25
end point density(μ^−1^)	134±5	548±101	266±41

**Table 2:** Avascular spaces (AVS) results (mean ± std dev).

Groups	Control	DSS	DSS+Sb
number of AVS	100±14	240±79	104±8
mean area (μm^2^) per AVS	9692±1747	3214±1169	6717±480
avascular area fraction (reciprocal of vascular area fraction, Table 1)	0.728±0.02	0.591±0.07	0.707±0.04

The reduction of angiogenesis in Sb-treated mice correlated with significant protection of the treated mice from DSS induced colitis as indicated by their weight loss and histological scores compared to control mice (Figure 1A and 1B). Together these data indicated that Sb could have a novel function in the regulation of angiogenesis during inflammatory responses leading to reduced tissue damage and allowing for faster mucosal recovery.

### Sb Inhibits VEGF-induced Angiogenesis in a Mouse Ear Neo-vascularization Model

**Figure 2:**
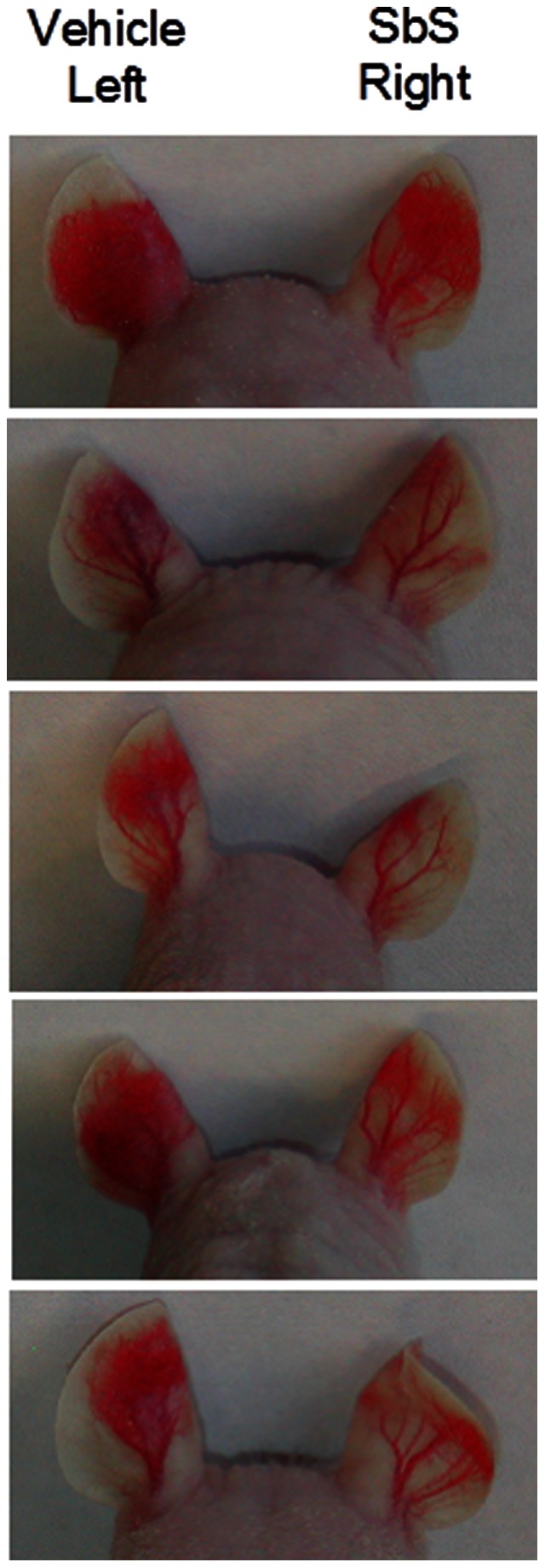
Sb inhibits VEGF-induced angiogenesis in the mouse ear vasculature model. The effects of Sb on VEGF-induced angiogenesis *in vivo* was assessed using an adenovirus expressing VEGF-A(164) (Ad-VEGF-A(164)) in the ears of adult nude mice (NuNu). Ad-VEGF-A164 (5×10^6^ pfu) was injected into both ears. One hour later the right ears of mice (N = 5) were injected s.c. with 10 µl SbS (ion-exchange chromatography enriched and DMEM diluted) and the left ears were injected s.c. with 10 µl vehicle (DMEM) as control. A second injection of SbS or vehicle was administered 24 hours later. Each of the 5 mice showed an angiogenesis response to Ad-VEGF-A164 in the control (left) ear. The inhibitory effect of SbS was evident with reduced new vessel formation in the right ear compared to the left. The images shown were obtained on day 16 after Ad-VEGF-A164 administration.

To determine whether Sb could directly regulate the function of VEGF, we then went on to assess the effects of Sb on VEGF-induced angiogenesis *in vivo* using an adenovirus expressing VEGF-A(164) (Ad-VEGF-A(164)) in the ears of adult nude mice (NuNu), an *in vivo* angiogenesis model as previously reported [35]. The right ears of mice (N = 5) were injected with 10 µl SbS (ion-exchange chromatography enriched and DMEM diluted) and the left ears were injected with 10 µl vehicle (DMEM) as control. The first treatment was administered 1 hour after 5×10^6^ pfu Ad-VEGF-A(164) was injected into both ears. The second SbS or vehicle injections were administered 24 hours later. As shown in Figure 2, each of the 5 mice showed an angiogenesis response to Ad-VEGF-A(164) in the control (left) ear. By day 7 an inhibitory effect of SbS was clearly evident with reduced new vessel formation in the right ear compared to the left. The effect of SbS remained evident until the end of the experiment on day 21. These results indicated that SbS mediated inhibition of vessel formation is mediated by a direct inhibition of VEGF mediated angiogenesis *in vivo*.

### *S. boulardii* Inhibits HUVEC Capillary Tube Formation

**Figure 3:**
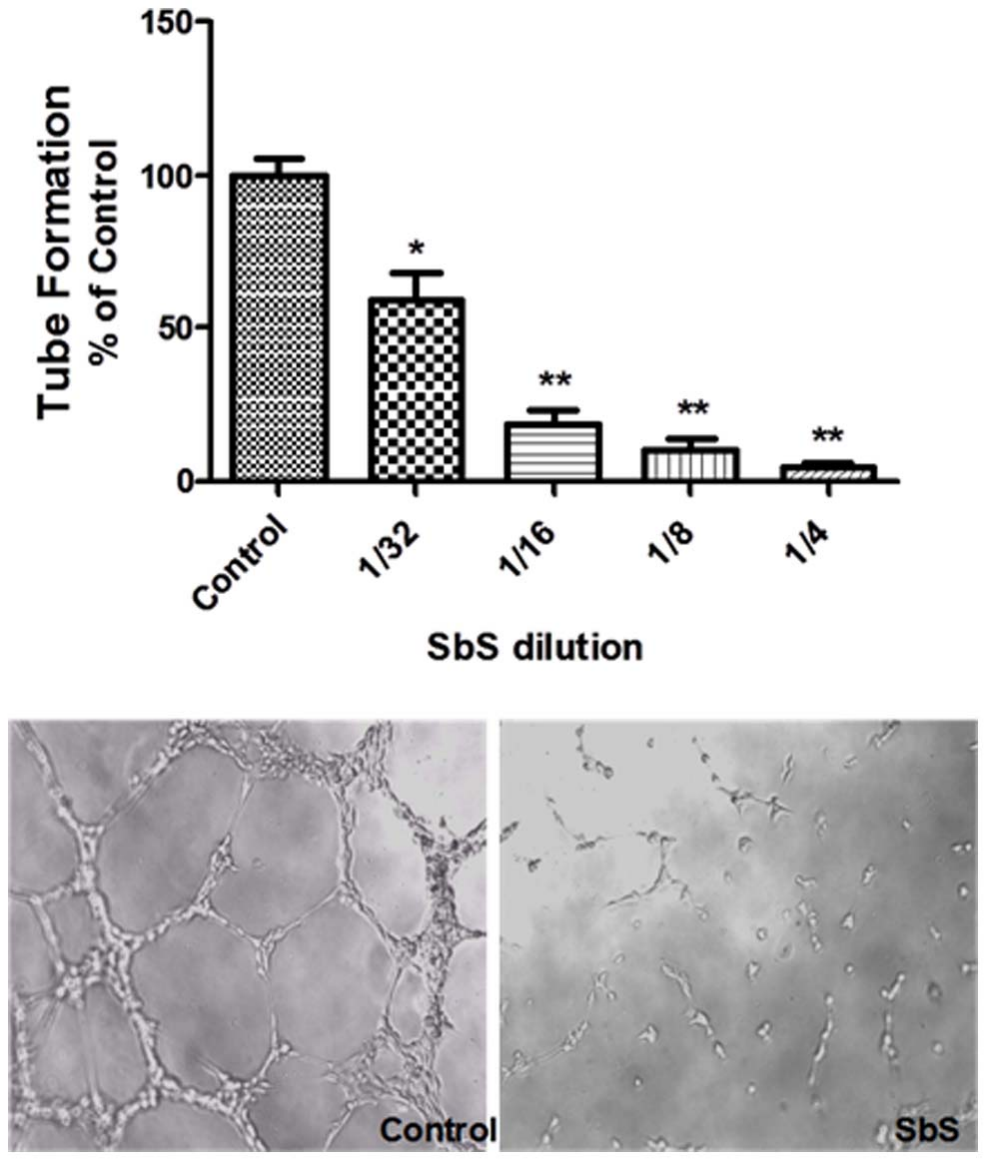
Sb inhibits HUVEC capillary tube formation. The ECMatrix™ in vitro angiogenesis assay was used to examine the effects of Sb on capillary tube formation. HUVEC (∼1×10^4^ cells) were cultured in 96-well plates previously coated with Matrigel and incubated for 16 hours at 37°C in the absence or presence of different doses of SbS. Representative images of tubule formation in the control and SbS-treated groups are shown in the lower panel. Tubular structures were counted from 10 randomly selected images. Percentage (%) of control is the mean number of tubules expressed as a proportion of that in the control group. SbS significantly inhibited HUVEC tubule formation in a dose-dependent manner (*p<0.05 for 1/32 dilution vs control; **p<0.01 for 1/16, 1/8, 1/4 vs control). Bars represent mean ± SE.

Endothelial cell capillary tube formation is a multi-step process involving cell adhesion, migration, differentiation and growth.[21] We used the ECMatrixTM *in vitro* angiogenesis assay to study the effects of SbS on angiogenesis *in vitro*. HUVEC (∼1×10^4^ cells) were cultured as triplicates in 96-well plates previously coated with Matrigel and incubated for 16 hours at 37°C in the absence or presence of SbS. Representative photomicrographs of tubule formation in the control and SbS-treated groups are shown in the lower panel of Figure 3. Tubular structures were then counted. Percentage (%) of control is the mean number of tubules expressed as a proportion of that in the control group. As shown in Figure 3, SbS significantly inhibited HUVEC tubule formation in a dose-dependent manner (P<0.05 for 1/32 dilution vs control; P<0.01 for 1/16, 1/8 and 1/4 vs control) indicating that, in addition to its effects on VEGFR signaling, SbS can attenuate angiogenic responses *in vitro*.

### *S. boulardii* Inhibits VEGFR Signaling in HUVEC

**Figure 4:**
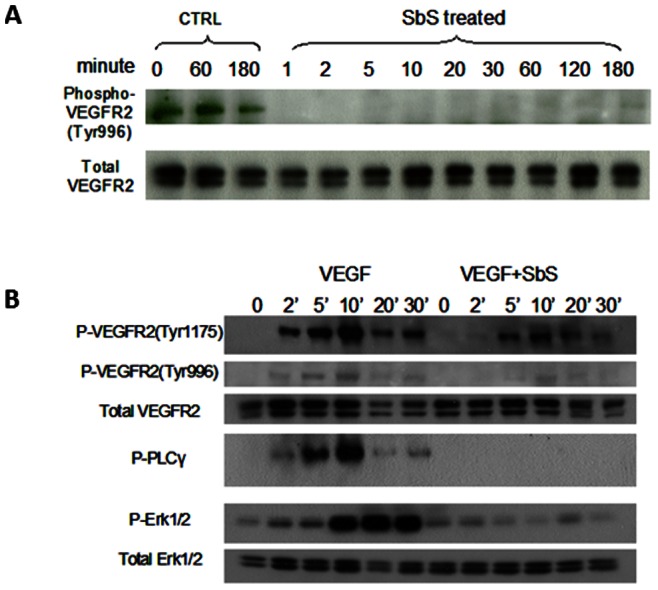
Sb inhibits VEGFR signaling in HUVEC. **4A)** HUVEC grown normally in complete medium were exposed to SbS for varying time periods (0–180 minutes). Cell extracts were prepared for Western blotting using antibodies against total and phopho-specific VEGFR-2. The band of phosphorylated VEGFR-2 immediately disappeared (within 1 minute) upon exposure to SbS. In contrast, the amount of total VEGFR2 remained stable. **4B)** HUVEC were starved overnight and then treated with 10 ng/ml VEGF in the presence or absence of SbS for 0 to 30 minutes. Cell extracts were prepared for Western blotting with antibodies against total or phopho-specific VEGFR-2, PLCγ, or Erk1/2. VEGF induced activation of both VEGFR-2 phosphorylation sites as recognized by p-Tyr1175 and p-Tyr996 phospho-site specific antibodies. The downstream mediators PLCγ and Erk1/2 are also activated by VEGF. The presence of SbS inhibited the phosphorylation of VEGFR-2 in response to VEGF and also reduced activation of the downstream kinases PLCγ and Erk1/2. Total protein levels were not affected.

VEGFRs belong to the same receptor tyrosine superfamily, as EGFR, IGF-1R, HER-2 and HER-3 which we have demonstrated to be a target of SbS in colonic epithelial cells [34]. We therefore examined the activation of VEGFR-2, the most defined and major mediator of angiogenesis, in HUVEC. Near-confluent (80% confluent) HUVEC grown in complete medium were exposed to SbS for varying time periods (0–180 minutes). Cell extracts were prepared for Western blotting using antibodies against total and phospho-specific VEGFR-2. As shown in Figure 4A, phosphorylated VEGFR-2 was evident under basal conditions and at 60 and 180 minutes of incubation. Phosphorylated VEGFR-2 disappeared immediately (within 1 minute) after exposure to SbS. In contrast, the amount of total VEGFR2 remained stable.

To test whether SbS inhibits ligand-induced VEGFR signaling, HUVEC (starved overnight) were treated with 10 ng/ml VEGF in the presence or absence of SbS for 0 to 30 minutes. Cell extracts were prepared for Western blotting with antibodies against total or phopho-specific VEGFR-2, PLCγ, or Erk1/2. As demonstrated in Figure 4B, VEGF induced activation of VEGFR-2 phosphorylation sites p-Tyr1175 and, less dramatically, p-Tyr996, within 2 minutes. The downstream mediators, PLCγ and Erk1/2 were also activated by VEGF as expected. SbS reduced VEGFR-2 phosphorylation in response to VEGF and also reduced activation of the downstream kinases PLCγ and Erk1/2. Total protein levels were not substantially altered. Together, these results demonstrate that SbS has the ability to inhibit VEGFR 2 signaling by preventing tyrosine phosphorylation required for the activation of PLCγ and Erk1/2 responsible for the proliferation of endothelial cells during vessel formation.

## Discussion

In this study, we propose a new mechanism by which *S. boulardii* regulates angiogenesis and controls mucosal inflammation. We demonstrate that Sb can inhibit VEGFR signaling and capillary tube formation in HUVEC. Sb can inhibit angiogenesis *in vivo* induced either directly by VEGF or by mucosal inflammation after epithelial barrier disruption by DSS.

For the first time, we were able to quantify vascular network structure and architecture in control and diseased intestines using several key parameters including vascular fractional area, fractional area of avascular spaces, and regularity of the geometry of avascular spaces, network morphology and the connectedness of the vascular network. Mapping and quantification by VESGEN showed that the uniform, lattice-like vascular network evident in control colonic tissues was greatly disrupted during DSS colitis. Inflammation greatly increased vessel density and disrupted the regular lattice geometry of the lamina propria microvasculature. We were also able to demonstrate that S *boulardii* treatment substantially enhanced mucosal recovery and restored the vascular network to a significant more normal morphology.

There is increasing evidence from experimental mouse models and from clinical observations that angiogenesis is an important component of IBD pathogenesis.[24], [29] For example, VEGF has been shown to be elevated in distal colonic tissue in the CD4+CD45RBhigh colitis model [22] as well as in intestinal tissues and sera of patients with Crohn’s disease and ulcerative colitis [39]–[41]. Recent studies in experimental colitis suggest that VEGF is an important mediator of IBD through promoting intestinal angiogenesis and inflammation [28]. Over-expression of VEGF in mice with DSS-induced colitis worsened their condition, whereas over-expression of soluble VEGFR, to block VEGF effects, had a beneficial effect [28]. Therefore, agents that inhibit VEGF/VEGFR signaling might prove to be useful to reduce intestinal inflammation in patients with IBD [24], [25].

Our work presented here is the first example of a probiotic possessing anti-angiogenic functions. However, *Bacillus polyfermenticus*, a prokaryotic probiotic strain, was reported topromote angiogenesis in the mucosa during recovery of mice from colitis [42]. Thus, considering probiotics as a single entity is likely to over-simplify their diverse and complex interactions with the intestine and its microbiota. On the other hand, angiogenesis may play different roles during the acute/injury and the recovery/healing stages of colitis.

*S. boulardii* appears to exert its therapeutic effect by multiple mechanisms and to influence several important facets of intestinal host-pathogen interaction, including neutralization of bacterial virulence factors [3], [43], enhancement of the mucosal immune response [44]–[46], interference with bacterial adhesion [3], strengthening of enterocyte tight junctions [6], altering immune cell redistribution [47] and modulating inflammatory signaling pathways of the host [33], [34], [48]. It will need to be established, whether the inhibition of vessel formation will result in a reduced recruitment of inflammatory macrophages, neutrophils or T cells, thus preventing severe mucosal tissue damage. The anti-angiogenesis property demonstrated in this study adds another novel mechanism of action for *Sb* and perhaps other probiotics.

In this study we report the exciting observation that probiotic yeast *S. boulardii* blocks VEGFR signaling and inhibits angiogenesis both *in vitro* and *in vivo*. Furthermore, this study demonstrates the enormous potential for probiotics to open new avenues toward understanding the pathogenesis of IBD as well as uncovering novel options for disease prevention and therapy.
